# Impact of High Salt-Intake on a Natural Gut Ecosystem in *Wildling* Mice

**DOI:** 10.3390/nu15071565

**Published:** 2023-03-23

**Authors:** Alessio Cardilli, Ibrahim Hamad, Aleksandra Dyczko, Sofie Thijs, Jaco Vangronsveld, Dominik N. Müller, Stephan P. Rosshart, Markus Kleinewietfeld

**Affiliations:** 1 VIB Laboratory of Translational Immunomodulation, Center for Inflammation Research (IRC), Hasselt University, 3590 Diepenbeek, Belgium; 2 Department of Immunology and Infection, Biomedical Research Institute (BIOMED), Hasselt University, 3590 Diepenbeek, Belgium; 3 Environmental Biology, Centre for Environmental Sciences, Hasselt University, Agoralaan Building D, 3590 Diepenbeek, Belgium; 4 Department of Plant Physiology and Biophysics, Institute of Biological Sciences, Maria Curie-Skłodowska University, 20-033 Lublin, Poland; 5 Experimental and Clinical Research Center, A Joint Cooperation of Max-Delbrück-Center for Molecular Medicine and Charité-Universitätsmedizin, 13125 Berlin, Germany; 6 Max-Delbrück-Center for Molecular Medicine in the Helmholtz Association (MDC), 13125 Berlin, Germany; 7 Charité–Universitätsmedizin Berlin, Corporate Member of Freie Universität Berlin and Humboldt-Universität zu Berlin, 13125 Berlin, Germany; 8 Department of Microbiome Research, Friedrich-Alexander-University Erlangen-Nürnberg, 91054 Erlangen, Germany; 9 Department of Medicine II, Medical Center—University of Freiburg, Faculty of Medicine, University of Freiburg, 79106 Freiburg, Germany; 10 University Multiple Sclerosis Center (UMSC), Hasselt University/Campus Diepenbeek, 3590 Diepenbeek, Belgium

**Keywords:** microbiome, high-salt diet, immunity, *wildling*

## Abstract

The mammalian holobiont harbors a complex and interdependent mutualistic gut bacterial community. Shifts in the composition of this bacterial consortium are known to be a key element in host health, immunity and disease. Among many others, dietary habits are impactful drivers for a potential disruption of the bacteria–host mutualistic interaction. In this context, we previously demonstrated that a high-salt diet (HSD) leads to a dysbiotic condition of murine gut microbiota, characterized by a decrease or depletion of well-known health-promoting gut bacteria. However, due to a controlled and sanitized environment, conventional laboratory mice (CLM) possess a less diverse gut microbiota compared to wild mice, leading to poor translational outcome for gut microbiome studies, since a reduced gut microbiota diversity could fail to depict the complex interdependent networks of the microbiome. Here, we evaluated the HSD effect on gut microbiota in CLM in comparison to *wildling* mice, which harbor a natural gut ecosystem more closely mimicking the situation in humans. Mice were treated with either control food or HSD and gut microbiota were profiled using amplicon-based methods targeting the 16S ribosomal gene. In line with previous findings, our results revealed that HSD induced significant loss of alpha diversity and extensive modulation of gut microbiota composition in CLM, characterized by the decrease in potentially beneficial bacteria from Firmicutes phylum such as the genera *Lactobacillus*, *Roseburia*, *Tuzzerella*, *Anaerovorax* and increase in *Akkermansia* and *Parasutterella*. However, HSD-treated *wildling* mice did not show the same changes in terms of alpha diversity and loss of Firmicutes bacteria as CLM, and more generally, *wildlings* exhibited only minor shifts in the gut microbiota composition upon HSD. In line with this, 16S-based functional analysis suggested only major shifts of gut microbiota ecological functions in CLM compared to *wildling* mice upon HSD. Our findings indicate that richer and wild-derived gut microbiota is more resistant to dietary interventions such as HSD, compared to gut microbiota of CLM, which may have important implications for future translational microbiome research.

## 1. Introduction

The gut of mammals is colonized by a complex and diverse bacterial community, which together with the host creates a delicate symbiotic relationship [1,2]. This bacterial community exerts many functions useful to the host, including metabolic, immunomodulating and trophic functions [3,4,5,6,7] and the gut microbiota composition could change during life, in line with the specific needs and physiology of the host [1,8,9]. Many beneficial functions of gut health-promoting bacteria are mediated by anaerobic fermentation derived metabolites [10,11,12,13] and dysbiotic conditions could significantly affect host health [2,11,14,15].

The growing concern for lifestyle impact on health has led to an increased scientific interest in gut microbiota involvement and its translational implications [16,17]. Indeed, the gut microbiota is shaped by both extrinsic (e.g., lifestyle, diet and medical treatments) and intrinsic (e.g., host genetics, immune and metabolic regulations) factors [8,18,19,20]. It is generally recognized that extrinsic elements could elicit impactful effects, with diet as one of the main contributing factors in affecting the gut microbiota composition and function [1,2,21]. Western dietary components, such as high-salt intake, are known to have a negative impact on host homeostasis by affecting the immune system and altering the gut microbiota and disease [18,22,23,24,25,26,27,28,29,30,31,32,33,34,35,36,37]. In murine gut microbiota, high-salt diet (HSD) is associated with reduction of health-promoting bacteria notoriously known as producer of short-chain fatty acids (SCFA) such as *Lactobacillus* spp., *Bifidobacterium*, *Blautia* and *Faecalibaculum* [28,29,38,39,40,41], alongside an increase in the abundance of *Akkermansia*, another opportunistic SCFA-producer that has been shown to affect host immunity and disease in different model systems [42,43]. 

Murine animal models are frequently used to study how dietary factors could shape the gut microbiota, immune system and disease [29,44,45,46]. Although the use of conventional laboratory mice (CLM) is still a valid option for many studies, it sometimes fails to properly translate gut microbiota-focused applications [47,48,49]. For instance, immunological and metabolomics research in murine models of inflammatory bowel disease (IBD) and obesity were shown to poorly predict translational outcome of gut microbiota studies [50]. This could be due to many inherent differences in these model systems, such as different gut anatomy, genetics and physiology [16,50]. However, another problem of using CLM for studying microbiota-immune interactions is the domestication of gut bacterial composition in CLM, which is mirrored in reduction of the complexity and resilience of the CLM gut microbiota compared to wild mice [51]. The need for sanitized and controlled environments faces a reduced presence of potential pathogens and parasites, which is believed to consequently lead to a less “educated” immune system in CLM compared to wild mice [51,52,53]. To address this problem, the *wildling* murine model was developed by C57BL/6 mice-derived embryo transfer into wild mice to obtain a wild-derived gut microbiota, in order to overcome the translational issue of immunological-gut microbiota studies [54]. Recent studies involving this mouse model showed superior outcome in predicting translational value of experimental immunotherapies compared to CLM [54,55]. Moreover, *wildling* gut microbiota was more resistant and resilient to antibiotics treatment and high-fat diet compared to CLM, comparable to the more complex situation in humans [54,55]. However, despite the established effects of HSD on gut microbiota, immune system and various disease models in CLM, the effects of high-salt intake on a natural, wild-derived gut microbiota are unknown. In this study, we thus evaluated the effect of HSD on different gut bacterial ecosystem compositions and predictive functions of CLM in comparison to *wildling* mice.

## 2. Materials and Methods

### 2.1. Animals and Diet

Wild-type C57BL/6 mice (7–8 weeks old females, n = 20) were purchased from Charles River and housed in the animal facility of the University of Hasselt under standardized conditions. *Wildling* mice (C57BL/6 genetic background, males n = 12 and females n = 11) [54] were housed in the animal facility of UHasselt under standardized conditions. Animal studies were approved by the Ethical Committee on Animal Experiments (ECAE) Hasselt University (ID201618A4V1, ID202235). Mice were housed (4 mice/cage) in a temperature-controlled room (21–23 °C) with a 12:12 h light/dark light cycle. The following purified diets were purchased from Ssniff (Soest, Germany): 0.5% NaCl/control diet (E15430-04), 4% NaCl/HSD (E15431-34). For HSD, animals were fed with 1% NaCl in the drinking water in addition to E15431-34, as described in [28]. CLM mice were equally distributed between control group (n = 10) and HSD (n = 10). For the *wildling* mice, male and female individuals were also equally distributed in control and HSD dietary groups (6 males for control, 6 males for HSD, 5 females for control and 6 females for HSD).

### 2.2. DNA Extraction

Microbial DNA extraction was performed as described in [28], by using a modified protocol of the QIAmp Fast DNA Stool Mini Kit (Qiagen, Hilden, Germany). In brief, fecal pellets were added to a 2-mL Eppendorf containing 0.5 mm glass beads and 1.5 mL of lysis buffer (ASL) (Qiagen, Hilden, Germany). Bead-beating was used to perform mechanical homogenization of the pellets. Full extraction was performed according to the manufacturer’s protocol with minor modifications (prolongation of the proteinase K incubation time to 2 h at 70 °C). DNA concentrations were evaluated using a NanoDrop ND-1000 spectrophotometer (NanoDrop Technologies, Wilmington, DE, USA) and stored at −20 °C before 16S rRNA gene amplification.

### 2.3. 16S rRNA Gene Amplification and Sequencing

16S rRNA gene sequence was amplified by using a primer specific for the V4 region (F515/R806), as previously described [56]. Briefly, 25 ng of DNA was used per PCR reaction (30 μL) (KAPA HiFi HotStart ReadyMix, Roche, Basel, CH, USA) of initial denaturation for 30 s at 98 °C, followed by 25 cycles (10 s at 98 °C, 20 s at 55 °C, and 20 s at 72 °C). Reactions were performed in triplicate, pooled per sample and purified by a magnetic bead-based clean-up system (Agencourt AMPure XP, Beckman Coulter, Brea, CA, USA). Library preparation was performed by a limited-cycle PCR to obtain the indexed library using Nextera technology (Nextera XT Index Kit, Illumina, San Diego, CA, USA), followed by a second AMPure XP magnetic beads clean-up step. Indexed samples were then normalized to the same concentration of 4nM, pooled and sequenced on an Illumina MiSeq platform PE300 with a 2 × 300 bp paired-end protocol according to company protocol (Illumina, Inc., San Diego, CA, USA).

### 2.4. Processing and Statistical Analysis of 16S rRNA Gene Sequencing Data

Raw sequences were processed using a QIIME 2 [57] pipeline. After length and quality filtering (default parameters), reads were filtered and assigned into operational taxonomic units (OTUs) using DADA2 [58]. Taxonomic assignment was performed by the VSEARCH algorithm (https://github.com/torognes/vsearch; accessed on 9 November 2022) and the Silva database v128 (https://www.arb-silva.de/; accessed on 9 November 2022). The ASV table was then normalized by rarefaction at 6.147 depth so that every sample reached the plateau at the end of the rarefaction curve. Alpha-diversity was assessed using two different metrics: OTUs richness (Observed), Chao1, Shannon, Simpson, Inverse Simpson (InvSimpson) ecological indexes. For beta-diversity, Bray−Curtis dissimilarity, Jaccard similarity, Weighted and Unweighted UniFrac metrics [59] were calculated and plotted by Principle Coordinates Analysis (PCoA) to visualize the real distance between samples. In order to normalize the OTU count table, rarefaction was performed at depth of 6305 sequences per sample 100 times. The output obtained from the OTU taxonomy assignment, as a taxonomy table, was used to collapse the normalized OTU table into tables for the taxonomy levels L2 (Phylum), L5 (Family) and L6 (Genus). Statistical analyses were performed by using R (https://www.R-project.org/; accessed on 25 November 2022; Version 4.2.0). The R package “vegan” (Version 2.6-4) [60] was used to generate beta-diversity metrics in order to compare compositional differences of groups by PCoA or by principal component analysis (PCA). Packages and data separation were tested by permutation test with pseudo-F ratios (function “Adonis” in “vegan”). Separation in terms of beta diversity between groups was tested by Permutational Multivariate Analysis of Variance Using Distance Matrices (PERMANOVA, function “Adonis” in “vegan”), while differences for intra-groups dispersion were tested by Multivariate homogeneity of groups dispersions test (PERMDISP, function “betadisper” in “vegan”). Taxa that were not present in at least 4 samples were excluded from the analysis. 

Differences in term of taxa relative abundances were first evaluated with preliminary Kruskal-Wallis test between 4 groups and then further evaluated with Wilcoxon-test between following comparison pairs: CLM Control vs. CLM HSD, *wildling* Control vs. *wildling* HSD, CLM Control vs. *wildling* Control, CLM HSD vs. *wildling* HSD. For evaluation of taxonomic differences between *wildling* and CLM, Linear Discriminant Analysis Effect Size (LEfSe: https://huttenhower.sph.harvard.edu/galaxy/; accessed on 25 November 2022) was used to distinguish the main features at genus level [61]. LEfSe results were then shown as a bar graph, with Linear Discriminant Analysis (LDA) score threshold higher than 1.0. Whenever necessary, *p*-values of multiple comparisons were adjusted by the Benjamini–Hochberg method. A false discovery rate (FDR) ≤ 0.05 was considered as statistically significant: * *p* ≤ 0.05; ** *p* ≤ 0.01; *** *p* ≤ 0.001. 

Functional differences between microbiomes of different NaCl content in the food (0.5% and 4% NaCl food content) were analyzed by PICRUSt2, a bioinformatics software package to predict metagenome functional content from 16s rDNA gene sequencing data (https://huttenhower.sph.harvard.edu/picrust/; accessed on 29 November 2022; PICRUSt2 2.4.1) [62]. PICRUSt2 pipeline was applied to representative sequences and their abundance table from DADA2 by using standard parameters (https://github.com/picrust/picrust2/wiki/Full-pipeline-script; accessed on 29 November 2022). From the full pipeline output, metagenomic prediction for KEGG Orthology and MetaCyc pathways were built as tables, with predictive functions as rows and samples as columns, and used to compare gut microbiota functions in *wildling* and CLM upon HSD regime. Microbial community predictive functions that contributed the most to the variation between *wildling* and CLM by first (PC1), second (PC2) and third principal component (PC3) were selected for further analysis upon HSD consumption in the two models. The matrix with the predictive function abundances was then normalized, transformed in Centered Log Ratio (CLR) values and log2mean ratio calculated (HSD/Control) for both *wildling* and CLM. Finally, the log2mean ratios were compared between groups by Wilcoxon-test and plotted as cuneiform plot. Differences between groups were statistically compared in R software using Wilcoxon-test and Kruskal-Wallis test functions and *p* values adjusted by the Holm or Benjamini–Hochberg method.

## 3. Results

### 3.1. HSD Affects Diversity and Composition of CLM and Wildling Gut Microbiota

**Figure 1:**
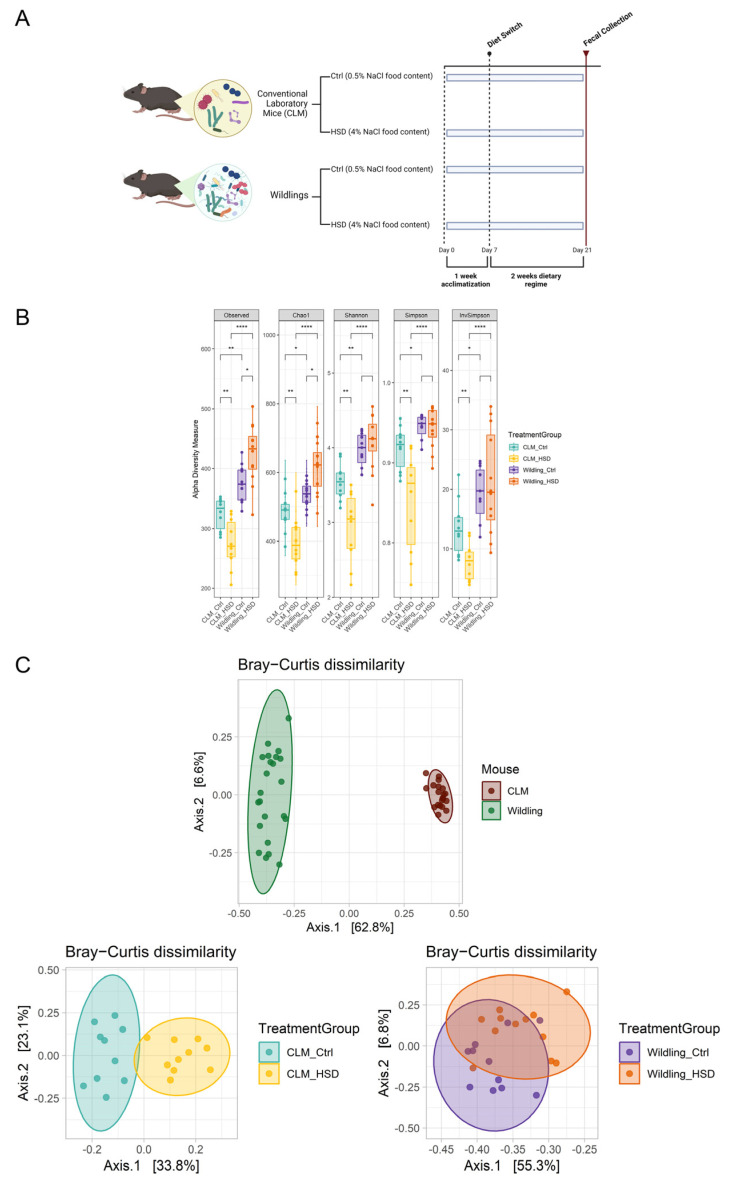
HSD impact on bacterial composition of CLM (n = 10/group) and *wildling* mice (n = 11 for *wildling* Ctrl and n = 12 for *wildling* HSD). (**A**) Experimental design. C57BL/6 CLM or *wildling* mice were fed on 0.5% NaCl (control, Ctrl) or high salt 4% NaCl (HSD) and gut bacterial community gut characterized by 16S rRNA gene amplicon sequencing. (**B**) Indexes for alpha diversity of fecal gut microbiota of CLM and *wildling*; from left to right, the following indexes are shown: Observed (OUT richness), Chao1, Shannon, Simpson, InvSimpson (Inverse Simpson). Differences between groups are evaluated statistically by Wilcoxon-test. (**C**) Principal coordinate analysis plot of beta diversity ordination from Bray−Curtis dissimilarity metric between CLM vs. *wildling* (top), CLM control vs. CLM HSD (bottom left) and *wildling* control vs. *wildling* HSD (bottom right); separation and homogeneity between groups was calculated by PERMANOVA and PERMDISP tests respectively. * *p* ≤ 0.05; ** *p* ≤ 0.01; **** *p* ≤ 0.0001.

To investigate the impact of HSD on a wild-derived gut microbial ecosystem in mice, we fed HSD or control diets to *wildling* mice and CLM. Mice were kept on dietary regimes for two weeks and the fecal gut microbiota composition was subsequently investigated by 16S RNA gene sequencing from fecal pellets collected at day 14 (Figure 1A). In line with a previous report, no strong differences were detected in terms of body weights between control and HSD groups of CLM and *wildling* mice [29]. 

To assess the different gut microbiota between the two models CLM and *wildling* mice at baseline, we estimated alpha diversity (Observed or Richness, Chao1, Shannon, Simpson and Inverse Simpson indexes), beta diversity (Bray−Curtis dissimilarity) and the main taxonomic differences. In line with previous studies [54], *wildling* gut microbiota was characterized by greater microbial richness (Figure 1B, all alpha diversity indexes), as well as a distinct and more heterogeneous microbial composition than CLM (Figure 1C, PERMANOVA p =0.001 & PERMDISP p = 0.0009, *wildling* vs. CLM; and Figure S1). In terms of microbial signatures, CLM and *wildling* mice gut microbiota were characterized by different bacterial taxa (Figure S1). In line with Rosshart et al. [54], bacterial taxa from *wildling* mice belong to *Intestinomonas*, *Desulfovibrio*, *Tuzzerella*, *Oscillobacter*, *Orodibacter* and the pathogenic genus *Helicobacter,* which characterized the wild-derived non-domesticated profile of this model (Figure S1). 

HSD induced significant reduction in bacterial diversity (Figure 1B, all alpha diversity indexes) as well as significant microbial shift in composition of CLM (Figure 1C, PERMANOVA *p* = 0.001, PERMDISP *p* = 0.1, CLM Ctrl vs. CLM HSD). In contrast, gut microbiota of *wildling* mice was characterized by higher diversity upon HSD (Figure 1B, Observed & Chao1 indexes), divergently from CLM, and they were also characterized by less pronounced microbial composition shift upon HSD compared to CLM (Figure 1C, PERMANOVA *p* = 0.001, PERMDISP *p* = 0.5, *wildling* Ctrl vs. *wildling* HSD).

### 3.2. Gut Microbial Composition of Wildling Mice Is More Resistant to HSD than CLM 

**Figure 2:**
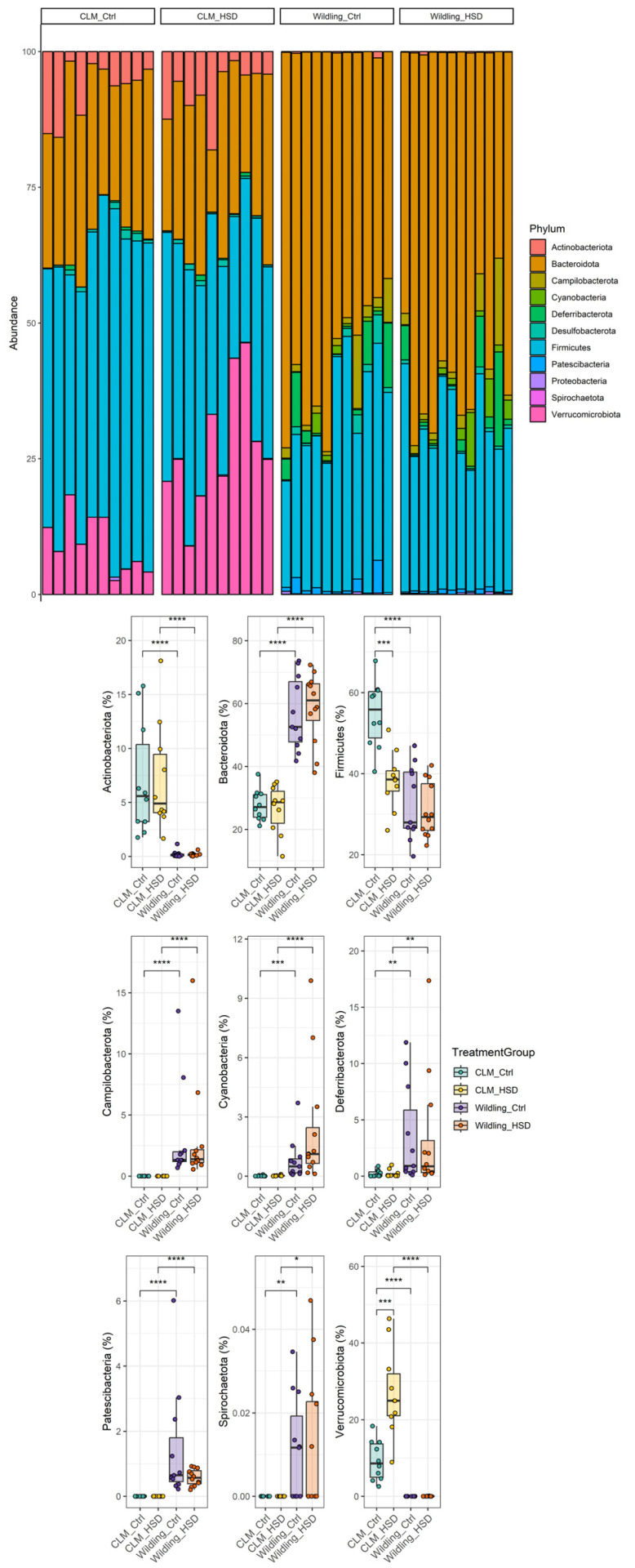
HSD-effect on bacterial phyla from gut microbiota of CLM (n = 10/group) and *wildling* mice (n = 11 for *wildling* Ctrl and n = 12 for *wildling* HSD). Total composition in terms of phyla relative abundance is shown by bar plot per each individual (top) and boxplot for specific phyla (bottom); statistical comparisons were performed between groups by Wilcoxon-test. * *p* ≤ 0.05; ** *p* ≤ 0.01; *** *p* ≤ 0.001; **** *p* ≤ 0.0001.

**Figure 3:**
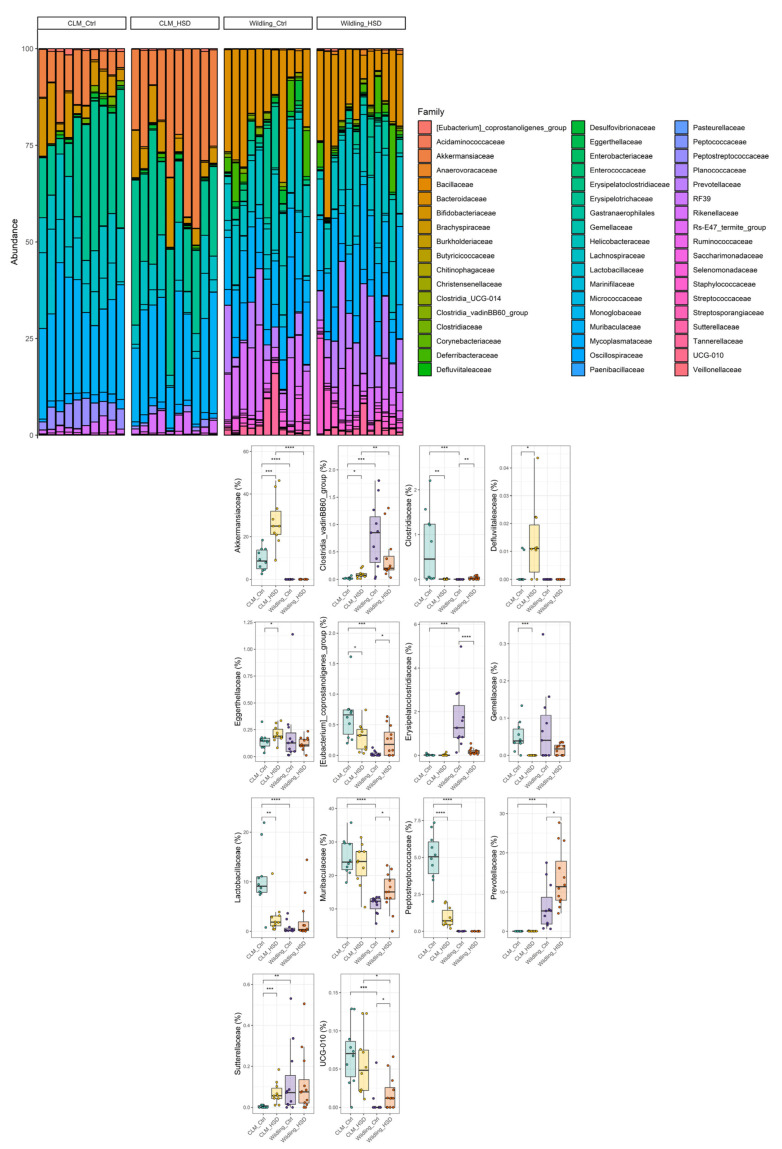
Impact of high-salt food consumption on bacterial families of CLM (n = 10/group) and *wildling* mice (n = 11 for *wildling* Ctrl and n = 12 for *wildling* HSD). Total composition at family level is represented by bar plot per each individual (on top) and boxplot for specific families (bottom); statistical comparisons were performed between groups by Wilcoxon-test. * *p* ≤ 0.05; ** *p* ≤ 0.01; *** *p* ≤ 0.001; **** *p* ≤ 0.0001.

**Figure 4:**
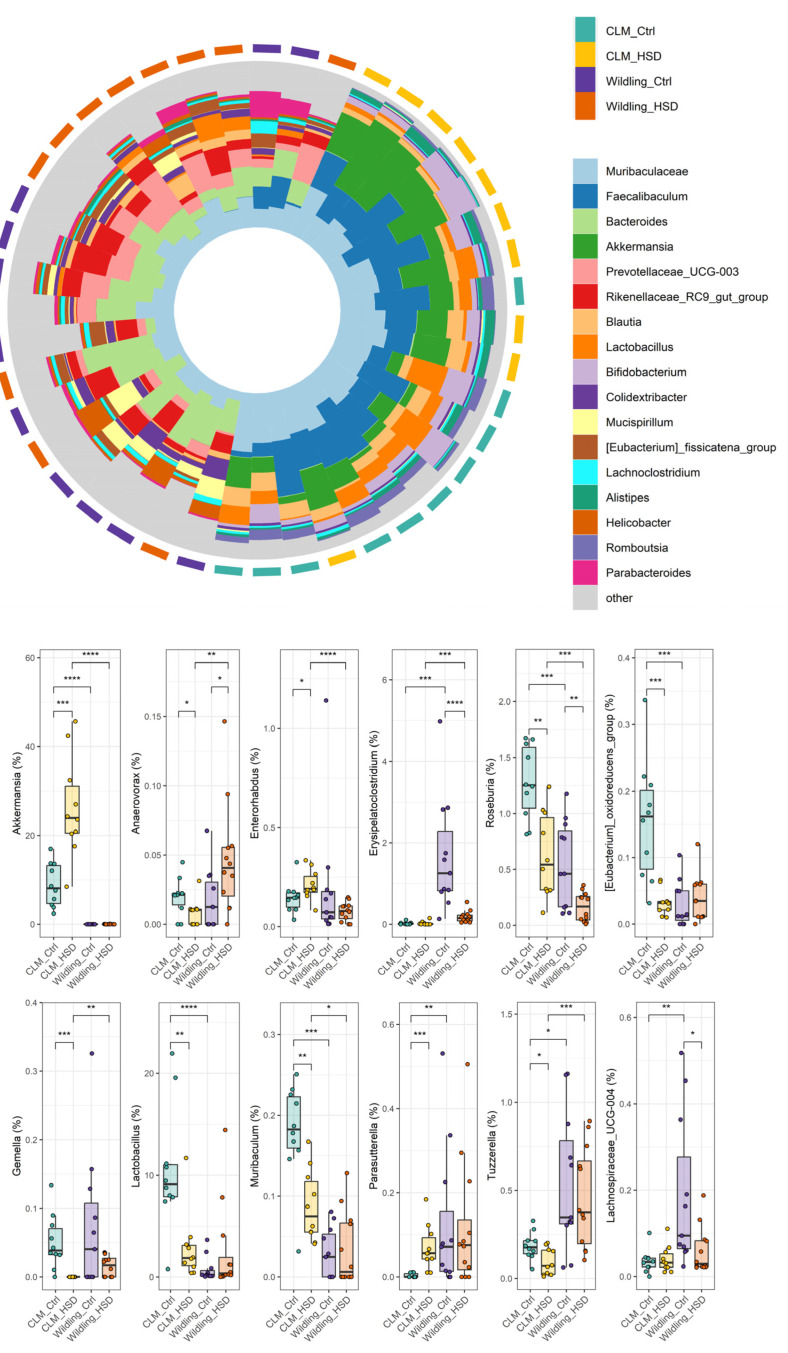
Changes in bacterial genera in CLM (n = 10/group) and *wildling* mice (n = 11 for *wildling* Ctrl and n = 12 for *wildling* HSD). Overall relative abundance contribution at genus level is plotted as circular bar plot per each individual (on top) and boxplot for specific genera (bottom); statistical comparisons were performed between groups by Wilcoxon-test. * *p* ≤ 0.05; ** *p* ≤ 0.01; *** *p* ≤ 0.001; **** *p* ≤ 0.0001.

Bacterial compositional differences between *wildling* and CLM were further taxonomically characterized. At the phylum level, the most abundant phyla in terms of relative abundance were: Firmicutes (CLM: 52 ± 12%, *wildling*: 32 ± 34%), Bacteroidota (CLM: 24 ± 23%, *wildling*: 57 ± 19%), Actinobacteriota (CLM: 10 ± 7%, *wildling*: 0.7 ± 1.3%) and Verrucomicrobiota (CLM: 24 ± 23%, *wildling*: 0%/not detected) (Figure 2). The gut microbial profile showed further different abundances for all phyla detected in fecal samples between *wildling* mice and CLM (Figure 2). Particularly, core microbiota phyla Firmicutes, Bacteroidota and Verrucomicrobiota were significantly different between the two models (Figure 2). More specifically, at the family level a different contribution was observed in *wildling* vs. CLM gut microbiota for most of the bacteria previously reported as HSD sensitive [28], including Lactobacillaceae, Clostridiaceae, Peptostreptococcaceae and Akkermansiaceae (Figure 3). In line with this, similar trends were confirmed at genus level between *wildling* and CLM samples for the main members of the forementioned families; among these, the most representative were *Lactobacillus*, *Roseburia*, *Tuzzerella*, *Faecalibaculum* and *Akkermansia* (Figure S1 and Figure 4). 

To characterize further the impact of HSD on CLM and *wildling* gut microbiota compositions, we also analyzed the impact of the dietary regimen at different classification levels. At the phylum level, HSD-treated CLM gut microbiota were characterized by significant depletion of Firmicutes and enrichment of Verrucomicrobiota (Figure 2), but none of the major phyla were affected by HSD in *wildling* samples (Figure 2). At the family level, CLM gut microbiota were characterized by significant depletion of lactic acid-producing bacteria such as Lactobacillaceae, as well as SCFA-producers such as Peptostreptococcaceae and Clostridiaceae (Figure 3). Additionally, in HSD-fed CLM, we observed increases in Akkermansiaceae, Sutterellaceae, Defluvitaleaceae and Eggerthellaceae (Figure 3). In contrast, HSD affected different bacterial families in *wildling* gut microbiota, among them the two highly-abundant Muribaculaceae and Prevotellaceae, both of which were increased upon HSD (Figure 3).

Bacterial modulation that most contributed to HSD-effect in CLM included the increase of genera *Akkermansia*, *Parasutterella* and *Enterorhabdus*, as well as the decrease of *Lactobacillus*, *Roseburia*, *Tuzzerella*, *(Eubacterium) oxidoreducens group*, *Muribaculum* and *Anaerovorax* (Figure 4). Except for *Roseburia*, none of the aforementioned genera were affected by HSD in *wildling* gut microbiota, while the genus *Anaerovorax* showed an opposite tendency from that of CLM (Figure 4).

### 3.3. HSD Affects Predictive Microbial Functions in CLM but Not in Wildling Mice

**Figure 5:**
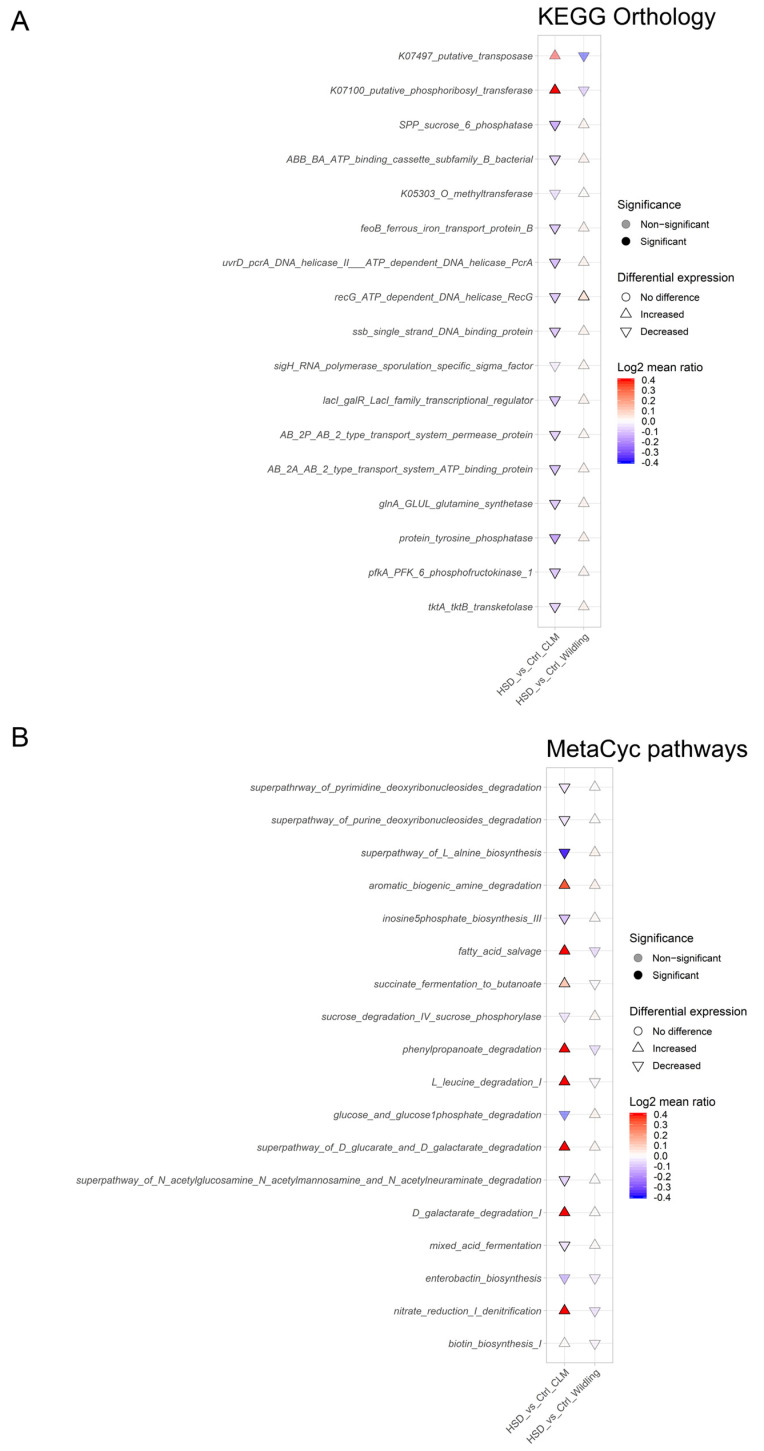
Effect of HSD on gut predictive metagenomic functions in CLM (n = 10/group) and *wildling* (n = 11 for *wildling* Ctrl and n = 12 for *wildling* HSD) gut microbiota. PICRUSt2 output plotted as cuneiform plot for KEGG Orthology annotation (**A**) and MetaCyc pathways (**B**) expressed as log2 mean ratio of the predictive functions counts between HSD vs. Ctrl samples. All statistical comparisons were performed between Ctrl vs. HSD groups by Wilcoxon-test.

PICRUSt 2 output did not detect any significant difference between microbial community functions of *wildling* HSD vs. untreated *wildling* mice for both KEGG Orthology and MetaCyc pathway annotations, with the only exception of HSD-induced increased function on *recG* gene for an ATP-dependent helicase from the KEGG Orthology (Figure 5A). HSD impact on CLM was characterized by significant decrease of predictive functions for KEGG Orthology, among them the gene *spp* (sucrose-6-phosphatase) and *pfkA* (phosphofructokinase 1), both involved in starch and sucrose metabolism, which is in line with previous findings [28] (Figure 5A). In addition, gut microbiota of HSD-fed CLM was characterized by decreased predictive functions of genes involved in membrane transport (*feoB* for iron transport, AB 2P AB 2 permease protein, AB 2A AB 2 ATP binding protein), glutamine biosynthesis (glnA), LacI family transcriptional regulator (*lacI*, *galR*) and transketolase (*tktA*, *tktB*) (Figure 5A). For MetaCyc pathways, HSD significantly enriched the CLM gut microbiota of predictive functions associated with nitrate reduction (denitrification pathway), galactose degradation (D-galactarate degradation, super pathway of D-glucarate and D-galactarate degradation), phenyl-propanoate degradation, fatty acid salvage, succinate degradation to butanoic acid and amino acid degradation (aromatic amine degradation, L-leucine degradation) (Figure 5B). Furthermore, in line with previous findings [28], HSD gut microbiota in CLM lost predictive functions for ammino acid biosynthesis (super pathway of L-alanine biosynthesis, L-lysine biosynthesis), mixed acid fermentation, with additional novel signature lost like N-acetylglucosamine/N-acetyl-mannosamine/N-acetylneuraminate degradation and deoxyribonucleosides degradation (pyrimidine and purine degradation, inosine5phosphate biosynthesis III) (Figure 5B).

## 4. Discussion

Complex and diverse *wildling* gut microbiota is known to be more resilient to certain disease models [51] and dietary regimes, such as high-fat intake [54,55]. However, no previous study has evaluated the effects of high sodium-intake on murine wild-derived gut microbiota. Here, we investigated for the first time how HSD affects *wildling* gut microbiota compared to CLM. Interestingly, our results demonstrated that, compared to CLM, the *wildling* microbiome is more resistant to HSD disturbance at both compositional and predictive functional levels. 

It is well established that high-salt intake could exacerbate risk of various diseases, such as cardiovascular or autoimmune diseases, by altering gut microbiome composition and immune homeostasis [25,29,31,34,63,64,65]. In line with previous reports, HSD-induced shifts in gut microbiota in CLM were characterized by significant alterations of microbial diversity, composition and predictive functions [28]. Health-promoting bacteria such as the Peptostreptococcaceae family and genera *Lactobacillus*, *Roseburia* and *Tuzzerella* decreased in terms of relative abundance in CLM, while *Akkermansia* significantly increased in HSD-fed groups. We also detected higher relative abundances upon HSD in Defluvitaleaceae, *Enterorhabdus* and *Parasutterella*. Interestingly, the genus *Parasutterella* is a core component of the gut microbiota of both CLM and humans, where it behaves as an asaccharolytic and producer of succinate [66]. Both *Enterorhabdus* from the Eggerthellaceae family and *Parasutterella* from the Sutterellaceae family are known to be enriched in patients with IBD [67,68], further indicating how HSD may affect disease development. However, and interestingly, *wildling* mice did not show a similar entity of HSD-induced microbial shifts, such as CLM. Despite this, *wildling* diversity significantly increased on HSD for observed OTUs and Chao1 metrics, and only a few taxa were involved in the HSD disturbance of *wildling* gut microbiota, among them an increase of *Anaerovorax*, coupled with a decrease of *Erysipelatoclostridium*, *Roseburia* and *Lachnospiraceae UCG-004* genus. *Roseburia* was the only bacterial signature commonly shared between HSD groups compared with the corresponding controls, despite HSD-fed CLM still being characterized by a higher abundance of this bacteria compared to HSD-fed *wildling* mice. Of note, butyrate-producing bacteria such as *Roseburia* were shown to have lower relative abundance in patients with ulcerative colitis [69] and this reduction was also observed to be correlated with IBD genetic risk of human subjects [70]. This is in line with previous findings, where shifts in bacterial genera such as *Roseburia* or *Lactobacillus* were found to be associated with risk of hypertension, possibly promoted by a western diet [71]. The bacterial composition of the gut is also associated to gut motility and physiology [72]. The genus *Anaerovorax* has previously been observed in mice with abnormal gut physiology and reduced motility [73]; however, the enrichment of *Anaerovorax* in HSD for *wildling* mice may lead to a different role of this taxa in the context of gut homeostasis and proper function. In line with previous findings, we observed an increase in the genus *Akkermansia* in the HSD group of CLM [28], while the gut microbiota of *wildling* mice was depleted of this genus, which is also consistent with earlier studies on this model [51,53,54,55]. Although the genus *Akkermansia* has been shown to be a potential probiotic due to its positive effect on improving host immunological and metabolic profiles (e.g., in obesity and type 2 diabetes) [42,74,75,76,77], the role of this genus is still unclear due to its negative correlation with clinical outcomes in colorectal cancer [78], Parkinson’s disease [79,80] and multiple sclerosis patients [81].

Consistent with our previous results obtained with MetaCyc pathways [28], CLM upon HSD showed decreased predictive microbial functions associated with starch and sucrose metabolism for KEGG orthology. However, the minor shifts in gut bacterial composition of HSD-fed *wildling* mice failed to induce any significant variations in predictive bacterial functions, indicating that *wildling*-derived gut microbiota and metabolic/ecological networks are much more stable and might adapt much more easily to HSD-induced dietary variations compared to CLM gut ecosystems, which warrants further investigation. Worth mentioning also is the possible influence of the gut fungal community on the gut bacterial network upon differential dietary regimes. Earlier studies have already suggested how potential interactions between bacteria and fungi are implicated in host immune system homeostasis and disease development [82,83,84,85]. In this context, CLM are further limited by their lower bacterial complexity compared to *wildling* mice, which may hinder the establishment of a diverse gut mycobiota [54]. Future studies will be able to determine the contribution of gut fungal communities in settings of gut microbiota and host immunity by using the *wildling* model.

In summary, our study provides data on how high sodium-intake affects a natural, wild-derived gut microbial ecosystem in comparison to a domesticated gut bacterial community of CLM. Our study demonstrated that HSD does not affect bacterial taxa and gut microbiota in *wildling* mice in the same way as it does for a domesticated gut microbiota from CLM. This divergence, as previously stated for other dietary regimens or conditions such as high-fat diets [54,55], indicates that future research is needed in natural murine model systems to recapitulate and to estimate the impact of dietary interventions on more complex gut ecosystems, as in humans. 

## Data Availability

The data sets used and/or analyzed during the current study are available from the corresponding author upon reasonable request.

## Supplementary Materials

The following supporting information can be downloaded at: https://www.mdpi.com/article/10.3390/nu15071565/s1, Figure S1: Major contributing bacteria to compositional differences between CLM (n = 10/group) and *wildling* (n = 11 for *wildling* Ctrl and n = 12 for *wildling* HSD) gut microbiota. Linear Discriminant Analysis (LDA) effect size (LEfSe) analysis revealed significant bacterial differences in fecal microbiota between CLM and *wildling* mice.
