# Supplementary figures and images for: Impact of High Salt-Intake on a Natural Gut Ecosystem in Wildling Mice
Source: Nutrients. 2023 Mar 23;15(7):1565. doi: 10.3390/nu15071565 (PMC10096756; doi:10.3390/nu15071565)

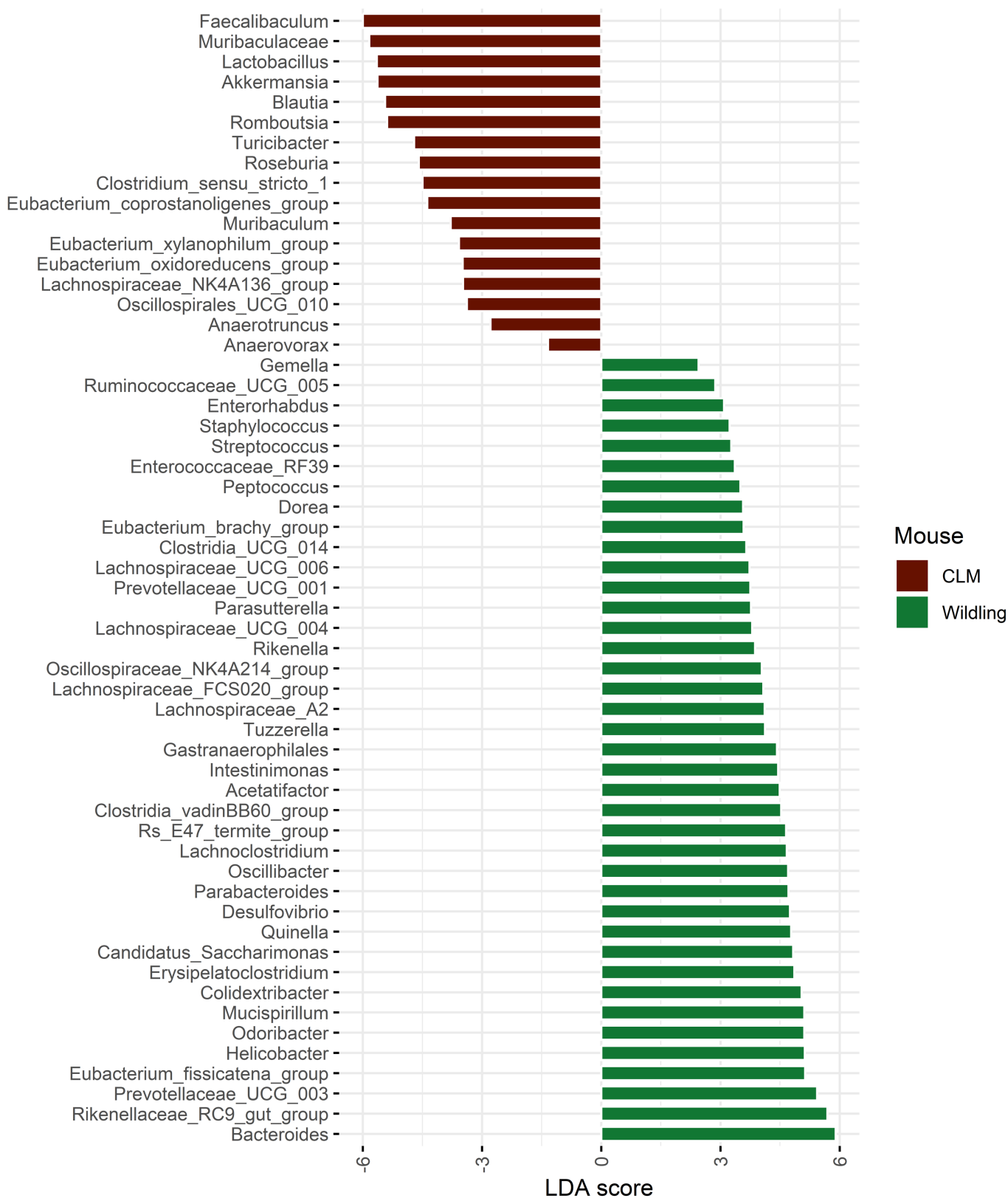

**Figure S1**

Supplement: Supplementary file 1 [file nutrients-15-01565-s001.zip › nutrients-2190680-supplementary.pdf]
